# Dr. Barry, on Vaccina

**Published:** 1801-11

**Authors:** John M. Barry

**Affiliations:** Cork


### To Dr. BRADLEY.

DEAR SIR,

A CASE related by Mr. Clutterbuck, in the 30th No. of the Phyfical Journal, which proved fatal after the Vaccine Inoculation, induces me to fend you an account of a cafe with fimilar fymptoms, which occurred in my practice. In January laft I inoculated the child of Mr. E. Scot, who had been previoufly a remarkably healthy little girl, then five months old. The veficle formed at the arm in five days after the inoculation, and the difeafe obferved its ufual progrefs of extreme mildnefs, till the 12th day, when the fore arm was affected with violent inflammation extending from the bend of the arm literally to the tops of the fingers. The puftule was on the decline. On the following day the inflammation, without abating on the fore-arm and hand, appeared above the puftule, and reached from thence over the fhoulder to the middle of the back. The glands under the arm were much fwelled, and the infant evidently fuffered confiderable pain. On the 16th day the inflammation fubfided on the fhoulder and back, leaving an effufion under the fkin of a dark colour. On the firft and fecond day of the inflammation I tried warm fomentations, which not being of the leaft fervice, I applied cloths dipped in aq. ammoniæ acetat. with apparent relief to the child, but without effecting any change in the fwellings. Mr. Ofborn, furgeon, was now called in, and recommended the application of leeches to the hand and arm, which were ftill occupied by the inflammation. Next day the rednefs difappeared, and the heat diminifhed, leaving the part ftill much fwelled. The inflammation now extended to the left fhoulder, and down the arm to the fingers, obferving the fame courfe as I have already defcribed on the right. In fome days the pudenda and thighs partook of the inflammation, which fucceffively appeared and difappeared feveral times on the arms. Leeches had been frequently applied, and once the blood flowed four hours from the bites of two of them, it being neceffary to ftop it by means of cauftic. In a fortnight from its firft appearance, the inflammation was entirely removed from every part of the body. The fever ftill, however, continued high, and the child was anafarcous from head to foot. The legs particularly were of a monftrous fize, and the water flowed from fome of the bites of the leeches, and fhe made very little urine in the day. To remove the anafarcous fwellings, I directed a weak folution of vegetable alkali, which in lefs than a week had the defired effect. She was, however, very weak and emaciated, and a new ſymptom of a very dangerous nature now occurred. The mouth aud throat were covered with aphthæ, ſo as nearly to obſtruct the paſſage, and to prevent the child from ſucking, which hitherto, even at the worſt period of her illneſs, ſhe had not ceaſed to do. By ſyringing the throat with wine and water and decoction of bark, and ſupporting the infant with wine whey and weak chicken broth, ſhe alſo got the better of this alarming ſymptom; and, after the burſting of two abſceſſes which had formed, one on each elbow, ſhe at length completely recovered, after two months of extreme anxiety to the parents, and of uncommon ſuffering to the child.

I have in this caſe detailed the practice as well as the ſucceſſion of ſymptoms, making no apology for the diſcordant nature of the medicines, in a complaint of which I was, till now, totally ignorant. From the ſtate of the pulſe, the great increaſe of heat in the ſyſtem, and all the ſymptoms taken together, I am diſpoſed to conſider this affection as highly inflammatory. I therefore infer, that the bleeding with leeches was very neceſſary, as it was undoubtedly the only practice which had any effect in removing the inflammation. Allowing this, however, I muſt confeſs, that the bleeding was puſhed too far; and previouſly to my having ſeen Mr. Clutterbuck's caſe, I attributed the general anaſarca to this cauſe, which was rather the effect of accident than deſign.

The caſes related by Mr. Maddock in your 24th Number, differ from Mr. Clutterbuck's, and that I have related, merely in the inflammation having commenced earlier, *viz.* about the time when the areola uſually forms about the inciſion, and extending from the inciſion; whereas, in the caſes I have related, as well as Mr. Clutterbuck's, the inflammation commenced beyond the inciſion, and the part occupied by the areola.

It would be of great advantage if gentlemen would publiſh the account of their practice, whether ſucceſsful or otherwiſe; as it is in this manner only that we can hope to arrive at the proper method of treating an affection, which, I am certain, is the only dangerous conſequence to be apprehended from Vaccine Inoculation. I hope this will be a ſufficient apology for occupying ſo much of your uſeful Journal with a ſingle caſe, the threatening aſpect of which, after Cow-pock Inoculation which I had practiſed on ſeveral hundreds without the leaſt danger, had occaſioned in me a mixture of ſurpriſe and uneaſineſs. I am,

Dear Sir, Yours, &c.

*Cork, Sept.* 23, 1801. JOHN M. BARRY.

